# Human Stem Cell-Derived GABAergic Interneurons Establish Efferent Synapses onto Host Neurons in Rat Epileptic Hippocampus and Inhibit Spontaneous Recurrent Seizures

**DOI:** 10.3390/ijms222413243

**Published:** 2021-12-08

**Authors:** Eliška Waloschková, Ana Gonzalez-Ramos, Apostolos Mikroulis, Jan Kudláček, My Andersson, Marco Ledri, Merab Kokaia

**Affiliations:** 1 Epilepsy Center, Department of Clinical Sciences, Lund University Hospital, 221 84 Lund, Sweden; ana.gonzalez_ramos@med.lu.se (A.G.-R.); apostolos.mikroulis@med.lu.se (A.M.); jan.kudlacek@lfmotol.cuni.cz (J.K.); my.andersson@med.lu.se (M.A.); marco.ledri@med.lu.se (M.L.); 2 Department of Physiology, Second Faculty of Medicine, Charles University, 150 06 Prague, Czech Republic

**Keywords:** human embryonic stem cells, GABA, interneurons, optogenetics, epilepsy, cell integration, synaptic integration

## Abstract

Epilepsy is a complex disorder affecting the central nervous system and is characterised by spontaneously recurring seizures (SRSs). Epileptic patients undergo symptomatic pharmacological treatments, however, in 30% of cases, they are ineffective, mostly in patients with temporal lobe epilepsy. Therefore, there is a need for developing novel treatment strategies. Transplantation of cells releasing γ-aminobutyric acid (GABA) could be used to counteract the imbalance between excitation and inhibition within epileptic neuronal networks. We generated GABAergic interneuron precursors from human embryonic stem cells (hESCs) and grafted them in the hippocampi of rats developing chronic SRSs after kainic acid-induced status epilepticus. Using whole-cell patch-clamp recordings, we characterised the maturation of the grafted cells into functional GABAergic interneurons in the host brain, and we confirmed the presence of functional inhibitory synaptic connections from grafted cells onto the host neurons. Moreover, optogenetic stimulation of grafted hESC-derived interneurons reduced the rate of epileptiform discharges in vitro. We also observed decreased SRS frequency and total time spent in SRSs in these animals in vivo as compared to non-grafted controls. These data represent a proof-of-concept that hESC-derived GABAergic neurons can exert a therapeutic effect on epileptic animals presumably through establishing inhibitory synapses with host neurons.

## 1. Introduction

Epilepsy is a neurological disorder affecting around 50 million people worldwide [1]. Patients suffering from epilepsy have access to a variety of symptomatic pharmacological treatments [2,3,4,5]. However, despite the growing number of these anti-seizure medications (ASMs), there are no established preventive or disease-modifying treatments available [6]. Moreover, long-term intake of ASMs is associated with adverse side effects [7,8,9], and most importantly, available medications are not effective in 30% of patients who become drug-resistant [10,11]. Most commonly these patients suffer from temporal lobe epilepsy (TLE), which is characterised by focal spontaneous recurrent seizures (SRSs) originating in the mesial temporal lobe often with secondary generalization. Many TLE patients also exhibit comorbidities, such as depression, anxiety, psychosis, and impairment of learning and memory [12]. For some of these drug-resistant patients, surgical resection of the epileptogenic focus may be an effective treatment, nonetheless, this therapeutic approach is possible only in a relatively small number of individuals due to the location of the seizure focus in, e.g., eloquent brain areas [13].

One of the structures affected in TLE is the hippocampus, where alterations of its neuronal circuitry lead to hyperexcitability [14]. One of the causes of hyperexcitability is dysfunction and/or degeneration of inhibitory GABAergic interneurons [15,16] which express and release the inhibitory neurotransmitter γ-amino-butyric acid (GABA) [17]. The loss of these neurons can lead to decreased inhibition in the neuronal networks, shifting the balance towards increased excitability, and reduced threshold for seizure initiation [18,19,20]. Therefore, there is an increased interest in developing cell therapies for epilepsy based on transplanting GABAergic progenitor cells in the seizure focus, thus enhancing inhibitory neurotransmission, which could normalize increased excitability of the local networks and thereby suppress SRSs. Several studies in animal models of TLE focused on medial ganglionic eminence (MGE)-derived GABAergic progenitor cells. After in vivo transplantation, these cells can differentiate into subclasses of interneurons typical for the hippocampus, migrate extensively, are capable of integration into the hippocampal circuitry, and most importantly, significantly diminish SRSs [21,22,23,24,25,26]. However, in all the studies, foetal rodent tissue has been used as the source of MGE-derived GABAergic progenitor cells. Although this approach provides a proof-of-concept for this idea, it lacks the translational potential for treating human patients. It is therefore not surprising that recent research has focused on the use of cells derived from human pluripotent stem cells (hPSCs), as a renewable resource for cell-based therapies. Studies using MGE-like GABAergic progenitors derived from hPSCs indicated seizure attenuation several months after transplantation in two rodent TLE models [27,28] suggesting that this strategy may be a promising approach for new therapy development.

In our previous study [29], we successfully generated optogenetically regulatable GABAergic interneurons from hESCs in vitro in a relatively short time, by adapting a protocol based on overexpressing two transcription factors, *Dlx2* and *Ascl1* [30]. Using optogenetic activation of these cells, we demonstrated the establishment of functional efferent synapses onto other human neurons in vitro [29]. In the current study, we asked whether these cells would also generate such synapses when grafted in vivo into the epileptic hippocampus. We transplanted these cells into the hippocampi of immunodeficient rats with kainate-induced TLE. We demonstrated that the hESC-derived GABAergic interneurons (hdInts) can functionally mature and form inhibitory synapses onto the host cells in the hippocampus already at three months and more prominently at six months post-transplantation (PT). Importantly, we observed a significant reduction of SRS frequency and total time spent in seizures in treated animals compared to untreated controls four months after status epilepticus (SE) induction. Taken together, our results provide evidence that hESC-derived interneurons suppress SRSs in epileptic animals by establishing inhibitory synaptic connections onto the host neurons and contribute to a better understanding of the potential mechanisms by which novel cell-based therapy would counteract refractory epilepsy.

## 2. Results

### 2.1. Kainic Acid-Induced Status Epilepticus and Development of Spontaneous Recurrent Seizures

**Figure 1:**
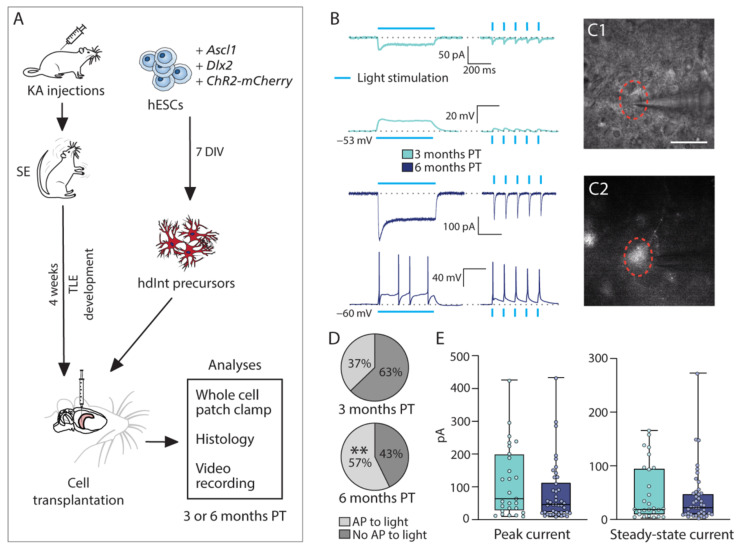
Experimental timeline and optogenetic properties of grafted hdInts. (**A**) Schematic representation of the timeline of the study. (**B**) Voltage- and current-clamp traces from ChR2-expressing hdInts indicate the direct response to 460 nm light stimulation. Two types of stimulation were used: continuous 500 ms pulse (left column) and brief 3 ms pulses at 10 Hz (right column). (**C1**) Grafted hdInt being approached by a patch-clamp pipette after identified as mCherry+ (shown on (**C2**); i.e., expressing ChR2). Red dotted circle outlines the patched cell. (**D**) Significantly higher proportion of cells was able to generate APs in response to light stimulation at six months PT. (**E**) Neither the peak current nor the steady-state current significantly changed over time. **, *p* < 0.01; Binomial test was used to compare proportions in (**D**); Mann-Whitney test was used to compare medians in (**E**). Scale bar 20 µm. KA, kainic acid; SE, status epilepticus; TLE, temporal lobe epilepsy; ChR2, channelrhodopsin-2; hESCs, human embryonic stem cells; DIV, days in vitro; hdInt, hESC-derived GABAergic interneuron; PT, post-transplantation; AP, action potential.

**Figure 10:**
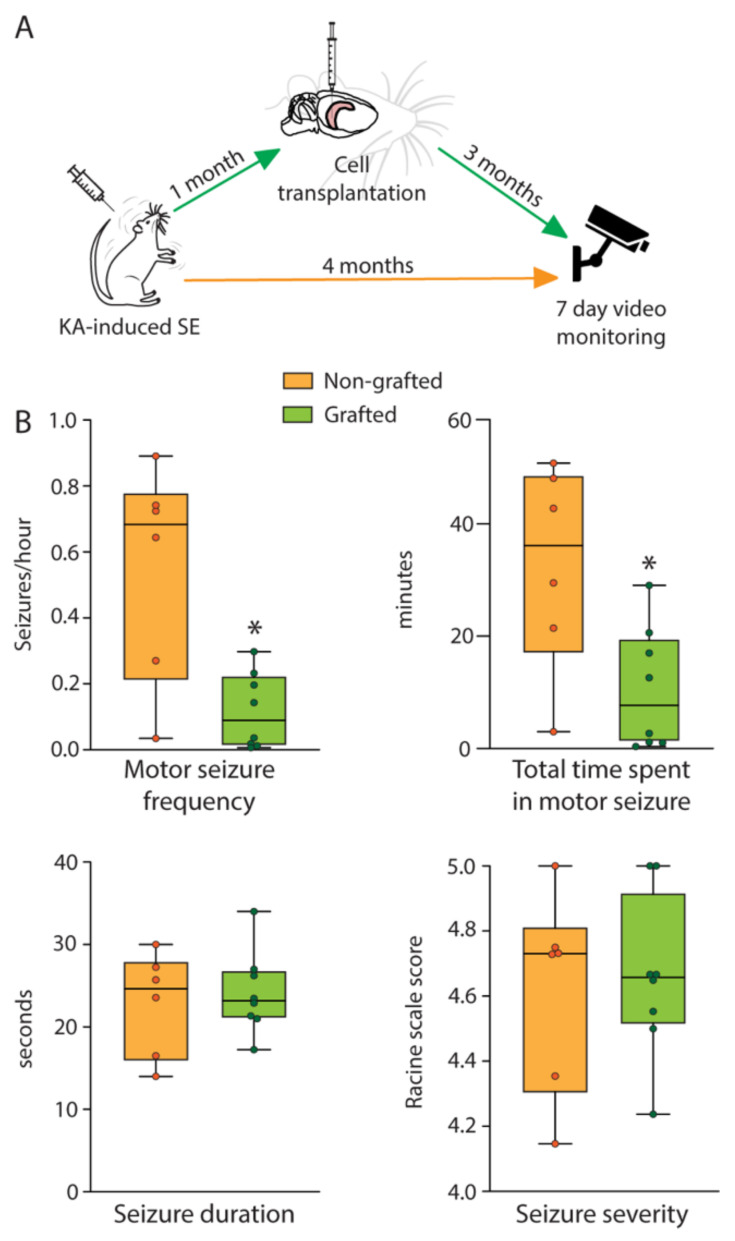
Effect of hdInt grafts on SRSs four months post SE. (**A**) Timeline of the experiments. ((**B**) upper panels) Graphs demonstrating median motor SRS frequency and total time in motor SRSs in control epileptic animals (non-grafted) and epileptic animals grafted with hdInts. ((**B**) lower panels) Graphs demonstrating median motor SRS duration and motor SRS severity in control epileptic animals (non-grafted) and epileptic animals grafted with hdInts. Note that motor SRS frequency and total time spent in motor SRS were significantly lower in the hdInt grafted group. *, *p* < 0.05. A Mann-Whitney test was used for comparison of medians in (**B**).

To assess kainic acid-induced SE (KA-SE), animals were observed since the first KA injection (Figure S1, Video S1). With a median dose of 15 mg/kg of KA, all animals developed SE (*n* = 25). The mortality rate for KA-SE was 8% (two out of 25 animals). Animals were divided in two groups, (i) non-grafted (controls; *n* = 8) and (ii) cell-grafted (*n* = 16). To establish the frequency of motor SRSs, eight animals from both groups were video monitored for seven days continuously, starting four months after SE (i.e., three months PT, Figure 1A and Figure 10A, Video S2). From the cell-grafted group, seven animals were used for electrophysiology and immunohistochemistry at three months PT, and eight animals were kept for six-month PT experiments. Motor SRSs were not detected in two rats from the control non-grafted group and were therefore excluded from the study. Video-EEG monitoring of three non-grafted rats used as a pilot, confirmed that 96.8% of electrographic SRSs were generalised motor SRSs (Figure S2A), therefore, only video recordings were used for the rest of the study to analyse SRS frequency and duration (Rat #1—16 motor seizures out of 17 total; Rat #2—17 motor seizures out of 18 total; Rat #3—27 motor seizures out of 27 total; Figure S2B1,B2).

### 2.2. hESC-Derived Interneuron Precursors Functionally Mature in the Epileptic Rodent Hippocampus

As described before, hPSC-derived interneurons can mature and integrate into the rodent hippocampus [27,28]. In this study, GABAergic interneuron precursors were generated from hESCs by overexpressing *Ascl1* and *Dlx2* [30] and were transplanted already at seven days in vitro (DIV; Figure 1A), with their differentiation continuing in vivo by administering doxycycline to the animals in the drinking water (see Section 4.3). Spare hdInt precursors from transplantation surgeries were replated directly on the same day and fixed 24 h after. Immunocytochemistry of hdInt precursors confirmed successful patterning of the cells (GABA+ and β-III-tubulin+, Figure S3D,E) and their loss of the proliferative state (Ki67-, Figure S3B). Cells did not express Sox2 (Figure S3A), a stem cell marker, nor Nestin, a marker of neural precursors at the stage of radial glia (Figure S3C). A more detailed characterization of hdInts in vitro was described in our previous study [29].

To be able to investigate the formation of synapses onto the host neurons, the grafted cells were transduced by channelrhodopsin-2 (ChR2)-carrying lentiviral particles. First, their ability to respond to blue light (460 nm) was assessed by whole-cell patch-clamp recordings. These experiments were performed both at three months PT (*n* = 27 cells) and six months PT (*n* = 42 cells, Figure 1B). The transplanted cells generated action potentials (APs) in response to the light, and the proportion of cells being able to generate APs was significantly higher at six months (*n* = 24 out of 42) compared to three months PT (*n* = 10 out of 27, *p* = 0.0098; Figure 1D, Table S1). However, the peak current of the response (3 months PT: 115.6 ± 20.97 pA, 6 months PT: 80.52 ± 13.98 pA), as well as the steady-state current (3 months PT: 50.23 ± 10.3 pA, 6 months PT: 41.50 ± 7.9 pA) did not differ between the two time points (*p* = 0.2287 and *p* = 0.8427, respectively; Figure 1E, Table S1).

**Figure 2:**
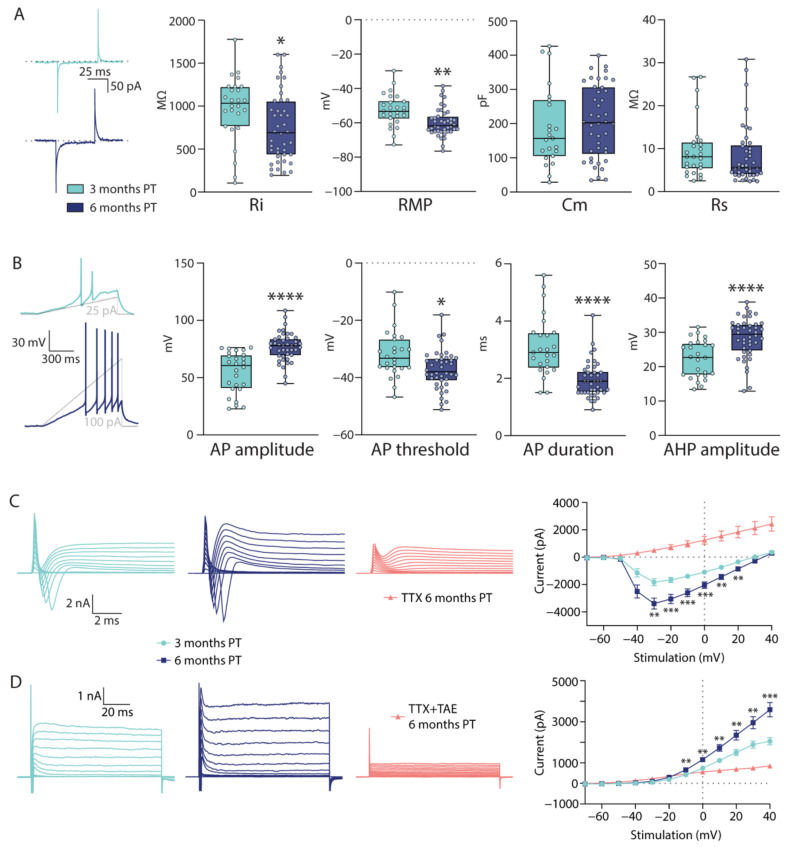
Electric membrane properties of grafted hdInts and their change over time. (**A**) Cell membrane properties of hdInts calculated from a series of 5 mV pulses applied through the patch pipette at three- and six-months PT. The Ri was lower and RMP higher, getting closer to mature neuronal characteristics at the later timepoint. (**B**) AP properties measured from induced APs. All changes between the two time points were significant and indicate neuronal maturation of the grafted cells. (**C**) TTX-sensitive evoked sodium currents were higher at six months PT. (**D**) TAE-sensitive evoked potassium currents were higher at six months PT. *, *p* < 0.05; **, *p* < 0.01; ***, *p* < 0.001; ****, *p* < 0.0001. Mann-Whitney test was used to compare medians in (**A**,**B**); Multiple unpaired *t*-tests were used to compare means in (**C**) and (**D**). Dotted grey line represents the baseline (0 mV/0 pA) from which reported values have been measured. Ri, input resistance; RMP, resting membrane potential; Cm, membrane capacitance; Rs, series resistance; AHP, after-hyperpolarisation; TTX, tetrodotoxin; TAE, tetraethylammonium.

Electrophysiological properties of the grafted cells were analysed to assess their functional maturation in the tissue environment of the epileptic hippocampus at three months PT (*n* = 27 cells) and six months PT (*n* = 42 cells). The input resistance (Ri) was significantly lower at six months PT (3 months PT: 974.0 ± 75.23 MΩ, 6 months PT: 746.4 ± 62.4 MΩ, *p* = 0.0164) while resting membrane potential (RMP) was significantly more negative at six months PT in the grafted cells (3 months PT: −52.64 ± 1.86 mV, 6 months PT: −59.77 ± 1.26 mV, *p* = 0.0016), better resembling the values of mature neurons (Figure 2A, Table S1). There was no significant difference in membrane capacitance (Cm, 3 months PT: 188.7 ± 24.01 pF, 6 months PT: 205.8 ± 16.37 pF, *p* = 0.4493) and series resistance (Rs, 3 months PT: 9.91 ± 1.35 MΩ, 6 months PT: 8.48 ± 1.08 MΩ, *p* = 0.1853; Figure 2A, Table S1). However, all AP properties showed a more mature neuronal phenotype at six months PT compared to the earlier time point, with the amplitude being larger (3 months PT: 54.56 ± 3.53 mV, 6 months PT: 76.79 ± 1.94 mV, *p* < 0.0001), the threshold lower (3 months PT: −31.82 ± 1.61 mV, 6 months PT: −36.5 ± 1.05 mV, *p* = 0.0134), the duration shorter (3 months PT: 3.11 ± 0.21 ms, 6 months PT: 1.95 ± 0.1 ms, *p* < 0.0001), and after-hyperpolarisation (AHP) amplitude larger (3 months PT: 22.28 ± 1.06 mV, 6 months PT: 28.39 ± 0.91 mV, *p* < 0.0001, Figure 2B, Table S1). All cells were able to fire depolarization-induced APs at both timepoints. In addition, a significant increase in sodium and potassium currents was observed at six months PT (Figure 2C,D). All the above-mentioned parameters indicate functional maturation of the grafted cells in the rat epileptic brain over time.

**Figure 3:**
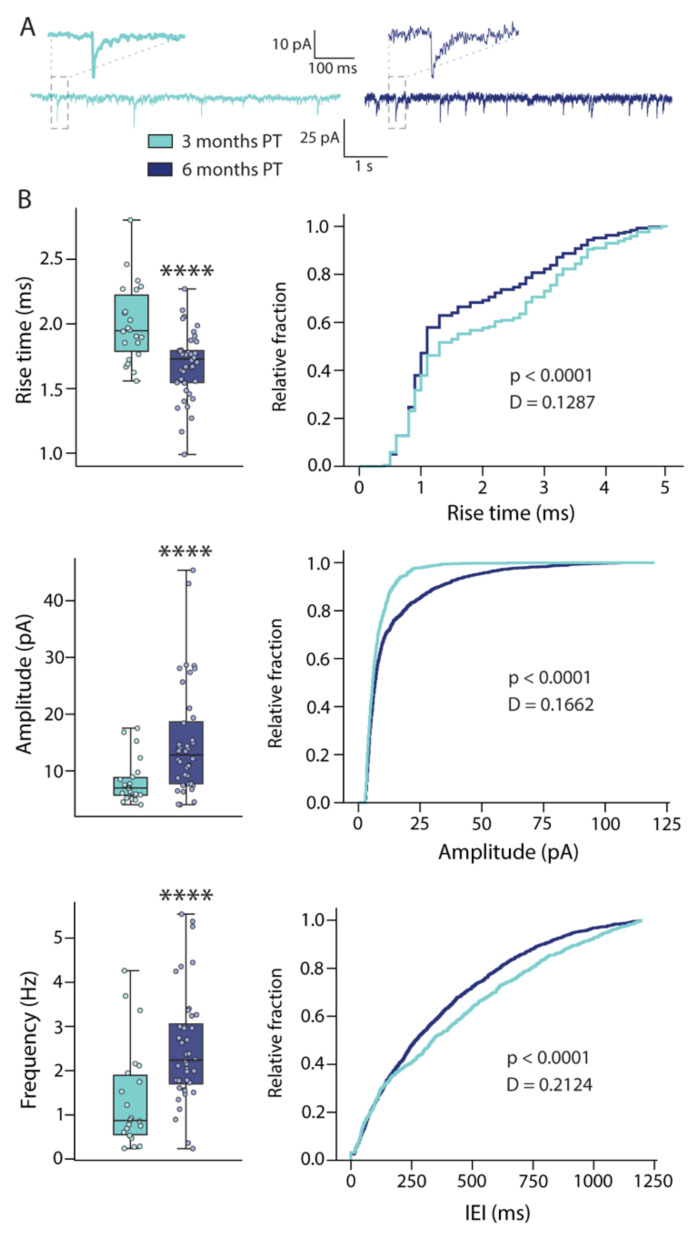
Spontaneous synaptic events in grafted hdInts. (**A**) Representative traces of voltage-clamp recordings from grafted hdInts in which sPSCs were detected. (**B**) Comparisons of sPSC characteristics between three- and six-months PT demonstrate the differences between medians and distributions. The rise time of the detected events is significantly shorter, amplitude significantly larger and frequency significantly higher at six months PT indicating improved synaptic integration at this later time point. ****, *p* < 0.0001. A Mann-Whitney test was used to compare medians in box plots, Kolmogorov-Smirnoff test was used for comparison of distributions. IEI, inter-event interval.

To investigate afferent synapses formed onto the grafted hdInts, we recorded spontaneous postsynaptic currents (sPSCs) in voltage-clamp mode at the holding potential of −70 mV (Figure 3A). sPSCs were then detected using a custom-made python script offline. The measured characteristics of the sPSCs were then compared between the cells at three- and six-months PT (*n* = 42 events/cell for distribution comparisons; for median comparisons: 3 months PT-*n* = 26 cells; 6 months PT-*n* = 42 cells). The median rise time of the detected events was significantly shorter at six months PT (3 months PT: 2.0 ± 0.06 ms, 6 months PT: 1.69 ± 0.04 ms, *p* < 0.0001), also clearly seen on the distribution curves (Figure 3B). Together with the significantly larger amplitude (3 months PT: 8.14 ± 0.77 pA, 6 months PT: 15.32 ± 1.6 pA, *p* < 0.0001), increased frequency (3 months PT: 1.36 ± 0.24 Hz, 6 months PT: 2.52 ± 0.2 Hz, *p* < 0.0001) and decreased inter-event intervals (IEIs) of the sPSCs at the later time point (Figure 3B), these changes indicate increased synaptic integration of the grafted hdInts into the host neural network over time (values summarized in Table S1).

### 2.3. hESC-Derived Interneurons form Inhibitory Synapses between Each Other and onto Host Neurons

**Figure 4:**
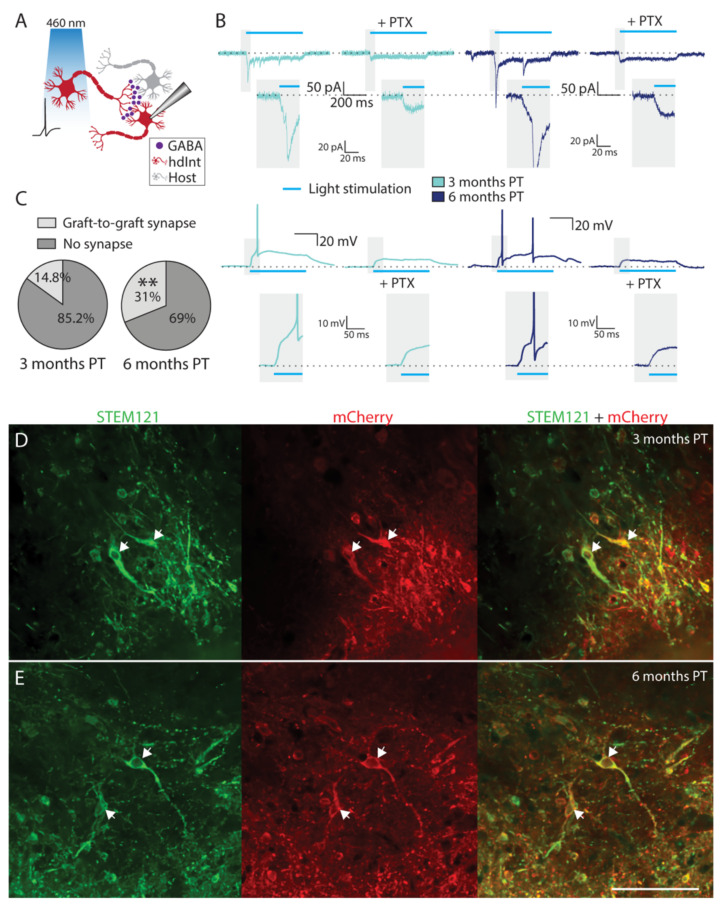
Graft-to-graft synaptic connectivity. (**A**) Cartoon representing a recording from a grafted hdInt receiving inhibitory synapses from another grafted hdInt (a graft-to-graft synapse) activated by blue light. (**B**) Representative traces from recorded hdInts in voltage- and current-clamp mode while illuminating slices by a 500 ms blue light pulse. Note a delayed synaptic response, which is then blocked by PTX, confirming the GABAergic nature of the synaptic connections. These PSCs were observed both at three- and six-months PT. (**C**) The proportion of cells with an observed graft-to-graft synapse was significantly higher at six months, compared to three months PT. (**D**,**E**) Immunofluorescent staining of slices used for electrophysiology experiments confirming the expression of mCherry in grafted hdInts. The cells exhibit staining for the human cell marker STEM121 (green) and mCherry (red). **, *p* < 0.01. The binomial test was used for the comparison of proportions in (**C**). Scale bar 100 µm. PTX, picrotoxin.

An important aspect of neuronal maturation and integration into the neuronal network is the ability of the transplanted cells to form efferent synapses onto the surrounding neurons. To investigate this, we took advantage of the optogenetic modification of the grafted hdInts. While recording from ChR2-expressing hdInts and illuminating the hippocampal slices with a 460 nm light pulse (Figure 4A), apart from the direct response to the light stimulation, delayed synaptic currents were also observed (with a delay from the light pulse onset; Figure 4B and Figure S5). The delayed onset of the PSCs was most likely due to the time required for the synaptic transmission, as well as the reach time needed for the depolarization of the presynaptic hdInt cells to the level of AP generation (Figures S6 and S7). To verify that the delayed currents were indeed inhibitory synaptic connections from surrounding hdInts, the same light stimulation recordings were performed by adding picrotoxin (PTX), a GABA receptor blocker. Indeed, the delayed currents were no longer visible after PTX treatment (*n* = 6 cells, Figure 4B), supporting the GABAergic nature of the graft-to-graft synapses. These graft-to-graft synapses were higher in proportion at six months PT (*n* = 13 out of 42 cells) compared to three months PT (*n* = 4 out of 27 cells, *p* = 0.0073) which suggests an increase in such synapses over time (Figure 4C). The ChR2-expression of grafted hdInts was identified before patching the cells by mCherry fluorescence and was confirmed retrospectively by staining against mCherry (Figure 4D,E).

**Figure 5:**
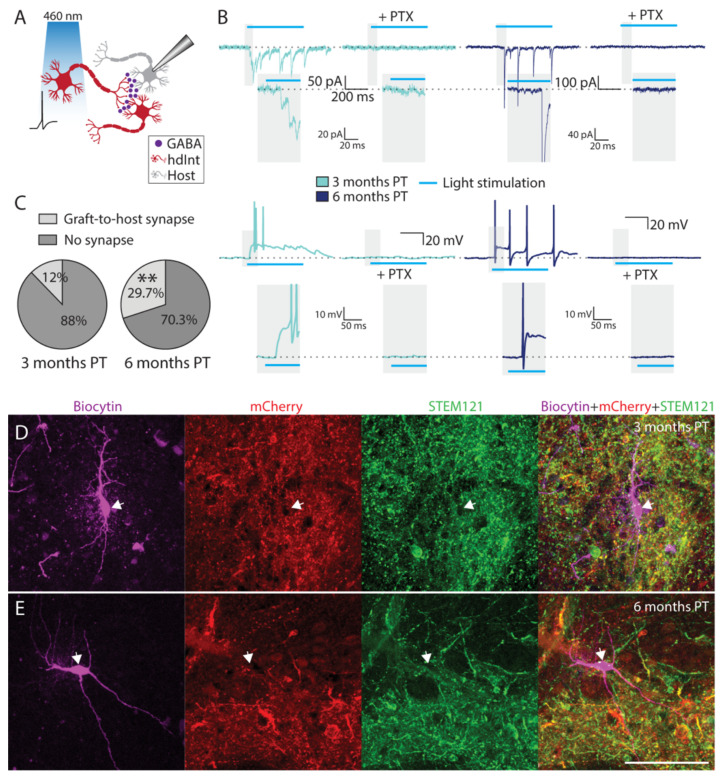
Graft-to-host synaptic connectivity. (**A**) Cartoon representing a recording from a host neuron receiving inhibitory synapses from a grafted hdInt (a graft-to-host synapse) activated by blue light. (**B**) Representative traces from recorded host neurons in voltage- and current-clamp mode while illuminating slices by a 500 ms blue light pulse. Note a delayed synaptic response, which is then blocked by PTX, confirming the GABAergic nature of the synaptic connections. These PSCs were observed both at three- and six-months PT. (**C**) The proportion of cells with an observed graft-to-host synapse was significantly higher at six months, compared to three months PT. (**D**,**E**) Immunofluorescent staining of slices used for electrophysiology experiments, the patched biocytin-filled cells (magenta) do not exhibit staining for STEM121 (green) nor mCherry (red). **, *p* < 0.01. A binomial test was used for the comparison of proportions. Scale bar 100 µm.

Similar experiments were performed to identify efferent synaptic connections from grafted hdInts onto the host neurons. Whole-cell patch-clamp recordings were performed from neurons not expressing ChR2 in the vicinity of a ChR2-expressing hdInt (Figure 5A). When a light pulse was delivered to the slice, there was no direct light-induced current observed (Figure 5B). However, in some cells delayed synaptic responses were detected instead (Figure 5B, Figure S5). These currents were readily blocked in all cells by PTX application (*n* = 5 cells), again confirming that the synaptic connections were GABAergic (Figure 5B). In contrast, no change was observed in the delayed synaptic responses when 2,3-dihydroxy-6-nitro-7-sulfamoyl-benzo-quinoxaline-2,3-dione disodium salt (NBQX) and (2R)-amino-5-phosphonovaleric acid (D-AP5) were applied to block glutamatergic synaptic responses (Figure S4). Similar, to the above-mentioned graft-to-graft synapses, the graft-to-host synapses were observed in a higher proportion of cells at the later time point (3 months PT: *n* = 3 out of 25 cells, 6 months PT: *n* = 11 out of 37 cells, *p* = 0.0032, Figure 5C). All recorded host neurons with delayed synaptic responses were retrospectively confirmed as host neurons by confocal microscopy, by not expressing mCherry nor the human cell marker STEM121 (Figure 5D,E).

To get a better understanding of the nature and origin of the delayed synaptic responses, time was measured from the onset of the light pulse to the base of the first delayed synaptic event. These measurements revealed a relatively narrow time window in which these events occurred in each cell (Figure S5). This finding indicates that the observed synaptic events were less likely to be random sPSCs but were rather delayed synaptic responses caused by light stimulation of a nearby ChR2-expressing hdInt. Notably, there was a quite wide range in the observed delayed synaptic responses between cells, especially at six months PT (Figure S5). To test whether one of the reasons could be a delayed AP generation in presynaptic hdInts after light onset, time was measured from the onset of the light pulse to the threshold of the first light-induced AP (only in cells with no graft-to-graft connections). A broad range of AP onset times was observed; thus a correlation analysis was performed which revealed that both light-induced peak current and steady-state current amplitudes correlated negatively with AP onset time (*r* = −0.7647, *p* = 0.0009; *r* = −0.7412, *p* = 0.0015, respectively; Figures S6A,B and S7).

### 2.4. hESC-Derived GABAergic Interneurons Express Interneuron Markers Calretinin and Calbindin

**Figure 6:**
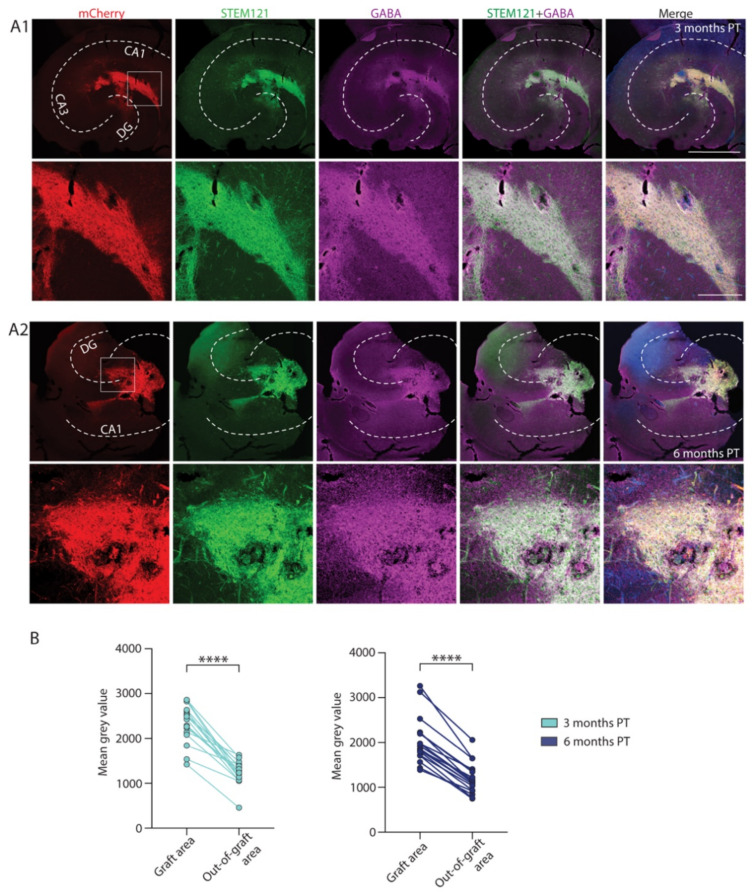
Histological analysis of GABA expression within and outside the graft. (**A**) Immunofluorescent staining of hippocampal sections with grafted hdInts for mCherry (marker for ChR2; red), human cytoplasm (STEM121; green), and GABA (magenta), merged with Hoechst (blue) in the last right column. GABA expression within the graft was well seen at both three months PT (**A1**) and six months PT (**A2**). (**B**) Quantification of GABA fluorescence intensity of the graft area compared with surrounding tissue at both timepoints. ****, *p* < 0001. Wilcoxon test was used in (**B**). Dashed white line in (**A1**,**A2**) represents the anatomical structure of the hippocampus. Scale bars 1 mm and 200 µm. DG, dentate gyrus.

The grafted hdInts have proven to be GABAergic in our previous in vitro study [29]. The present electrophysiological experiments suggested that even after in vivo grafting these cells become GABAergic as judged by their efferent synaptic connections. To further consolidate their phenotype, the hippocampal slices used for whole-cell patch-clamp recordings were used for immunohistochemistry. Firstly, the expression of GABA in the tissue was examined, and indeed, the grafted areas expressed significantly higher fluorescence levels of GABA staining than the surrounding tissue both at three months PT (*n* = 17 slices, *p* < 0.0001) as well as at six months PT (*n* = 19 slices, *p* < 0.0001; Figure 6A1,A2). Summarized data are presented in Figure 6B.

**Figure 7:**
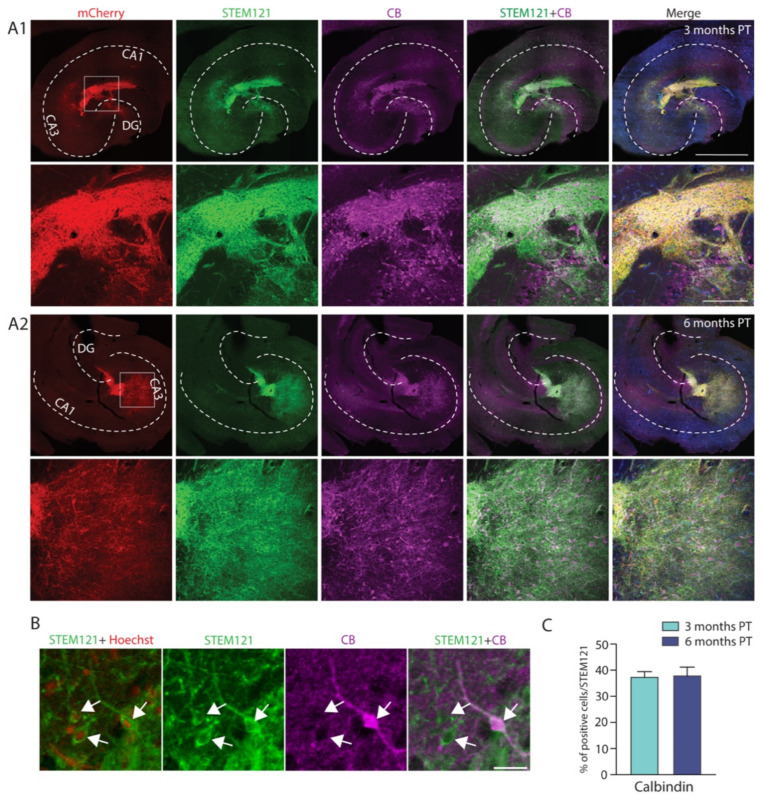
Grafted hdInts express calbindin, a marker of a GABAergic interneuron subpopulation. (**A**) Immunofluorescent images of hippocampal sections with grafted hdInts stained for mCherry (marker for ChR2; red), human cytoplasm (STEM121; green), and calbindin (CB; magenta), merged with Hoechst (blue) in the last right column. CB expression within the graft was observed at both three months PT (**A1**) and six months PT (**A2**). (**B**) A merged image of STEM121 and Hoechst was used for counting all STEM121+ cells, while the merged image of STEM121 and CB staining was used for counting STEM121+CB+ double-positive cells. Arrowheads indicate examples of counted cells. (**C**) Quantification of cells stained for STEM121 and CB. The percentage of cells co-expressing both CB and the human cytoplasm marker was similar at both timepoints. Unpaired *t*-test was used for comparison of means in C. Dashed white line in (**A1**,**A2**) represents the anatomical structure of the hippocampus. Scale bars: 1 mm ((**A1**,**A2**) upper row), 200 µm ((**A1**,**A2**) lower row), and 20 µm (**B**).

To further specify the interneuron phenotype, we examined slices for calretinin (CR) and calbindin (CB) immunoreactivity. These stainings revealed that the expression of CB in the grafted hdInts was colocalized with the expression of the human cytoplasm marker STEM121 in a proportion of cells (Figure 7A1,A2), which was similar at both time points of analysis (*n* = 21 slices/timepoint, three months PT: 37.48 ± 1.98%, 6 months PT: 38.0 ± 3.18%, *p* = 0.8904; Figure 7C). Examples of double labelled cells with CB and STEM121, as well as STEM121 and Hoeschst are presented in Figure 7B.

**Figure 8:**
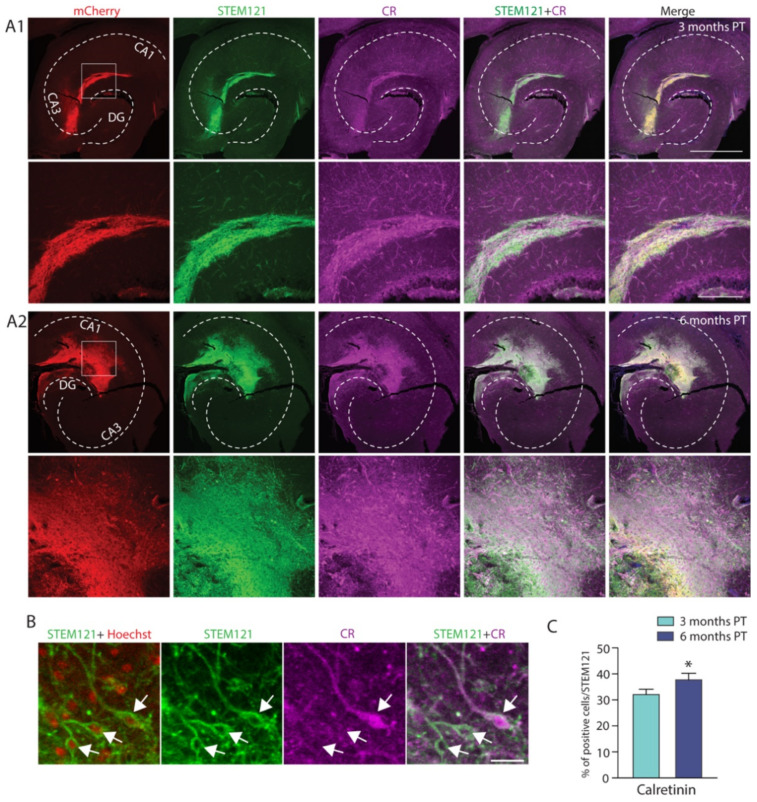
Grafted hdInts express the interneuron subpopulation marker calretinin. (**A**) Grafted hippocampal sections stained for mCherry (marker for ChR2; red), human cytoplasm (STEM121; green), and calretinin (CR; magenta), merged with Hoechst (blue) in the last right column. STEM121+ cells expressing CR were observed at both three months PT (**A1**) and six months PT (**A2**). (**B**) A merged image of STEM121 and Hoechst was used for counting all STEM121+ cells, merged image of STEM121 and CR staining was used for counting STEM121+CR+ double-positive cells. Arrowheads indicate counted cells. (**C**) Quantification of cells stained for STEM121 and CR. Note that the percentage of cells co-expressing both CR and the human cytoplasm marker was significantly higher at six months PT compared to three months PT. *, *p* < 0.05. Unpaired *t*-test was used for comparison of means in (**C**). Dashed white line in (**A1**,**A2**) represents the anatomical structure of the hippocampus. Scale bars: 1 mm ((**A1**,**A2**) upper row), 200 µm ((**A1**,**A2**) lower row) and 20 µm (**B**).

Cells expressing both CR and the human cytoplasm marker STEM121 were identified in the graft as well (Figure 8A1,A2), with the percentage of cells co-expressing both markers being significantly higher at the six-month PT timepoint as compared to three months PT (*n* = 21 slices/timepoint, three months PT: 32.42 ± 1.66%, 6 months PT: 38.07 ± 2.17%, *p* = 0.0455; Figure 8C). Examples of double labelled cells with CR and STEM121, as well as STEM121 and Hoeschst are presented in Figure 8B.

In summary, hdInt precursors grafted into the rat epileptic hippocampus mature into GABAergic interneurons mostly with a CB and CR phenotype.

### 2.5. Optogenetic Activation of Grafted hESC-Derived Interneurons Reduces the Rate of Epileptiform Discharges In Vitro

**Figure 9:**
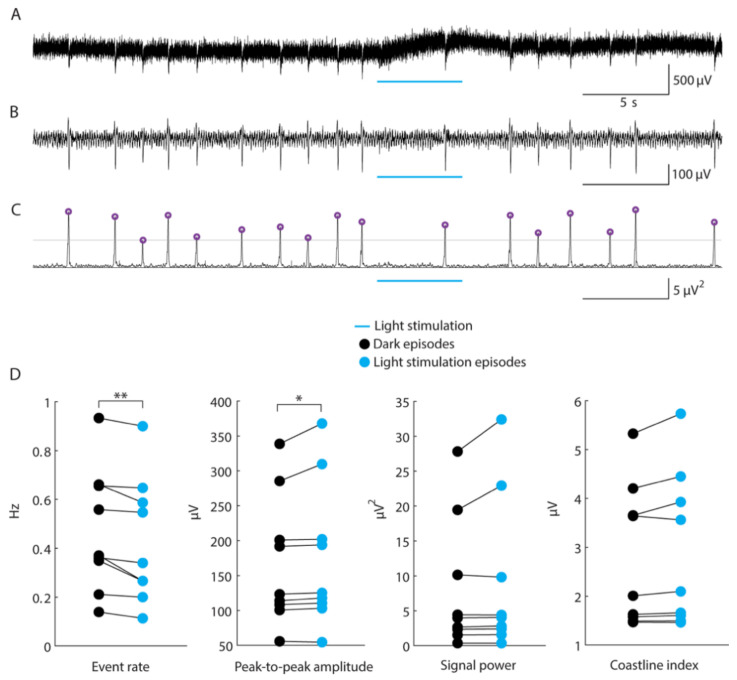
Light-induced activation of grafted hdInts reduces the rate of induced epileptiform discharges. (**A**) Example of a raw local field potential (LFP) recording with epileptiform discharges. (**B**) Filtered LFP trace. (**C**) Power of the filtered signal calculated in 50 ms windows shifted sample by sample. Grey horizontal line marks the detection threshold while purple circles mark the detections. (**D**) Parameters of the epileptiform discharges during dark periods and during light stimulations. *, *p* < 0.05; **, *p* < 0.01. The Wilcoxon signed-rank test was used for comparisons in (**D**).

To investigate whether GABA released from the synaptic terminals of transplanted hdInts may exert any effect on network activity in the hippocampus we took advantage of the ChR2 expression in these cells. A blue light was illuminated on the slices during ongoing pseudo-regular epileptiform discharges induced by high-K^+^ or zero-Mg^2+^ artificial cerebrospinal fluid (aCSF). Examples of the raw and filtered epileptiform discharges recorded in the slices and their detection rate are presented in Figure 9A–C. The mean frequency of these discharges was estimated as 0.47 Hz, peak-to-peak amplitude 168 μV, peak power 8.08 μV2, and coastline index 2.78 μV. The light pulse trains at 30 Hz frequency applied to the slices did not lead to any change in the epileptiform discharges. The 5 s continuous light exposure, however, slightly but statistically significantly reduced the rate of the epileptiform discharges by 0.041 ± 0.012 (−0.026) Hz and increased the peak-to-peak amplitude by 7.3 ± 3.7 (2.1) μV (*n* = 9 hippocampal slices, *p* = 0.0039, *p* = 0.0117, respectively). There was no change in the power and the coastline index (Figure 9D). These data show that GABA release from the transplanted neurons can interfere with ongoing synchronized epileptiform discharges, suggesting that they might have functional effects on seizure activity in vivo as well.

### 2.6. Grafting of hESC-Derived Interneuron Precursors Reduces Seizure Frequency in Epileptic Rats

To investigate whether these cells also inhibit SRSs in vivo, we analysed behavioural motor SRSs at four months post SE based on continuous seven-day video recordings of non-grafted and grafted rats (*n* = 8 rats/group, Figure 10A). Seizure frequency, total time spent in seizure, average seizure duration, and severity were evaluated (Figure 10B). Grafted animals showed a statistically significant decrease in the motor SRS frequency compared to the non-grafted group (median decrease 87%, *p* = 0.0200, Figure 10B, left panel). Furthermore, the grafted animals exhibited significantly shorter total time spent in motor seizures (10.53 ± 3.84 min) compared to controls (32.48 ± 7.47 min, *p* = 0.0123, Figure 10B, right panel). In contrast, neither average motor SRS duration (non-grafted: 22.83 ± 2.57 s, grafted: 24.14 ± 1.78 s, *p* > 0.9999) nor their severity (non-grafted: 4.62 ± 0.13 grade, grafted: 4.66 ± 0.09 grade, *p* = 0.8252) differed between the two groups (Figure 10B). It is worth noticing that this seizure-suppressant effect of transplanted hdInts was exerted without optogenetic activation of these cells.

In summary, these results indicate a seizure-suppressant effect of grafted hESC-derived GABAergic interneurons in rats with KA-induced TLE. This positive outcome is probably the result of the grafted cells integrating into the hippocampal network by forming inhibitory synapses and releasing GABA, which inhibits seizure activity in the epileptic network of the hippocampus.

## 3. Discussion

In the presented study, we demonstrate that the transplanted hESC-derived GABAergic neurons mature and integrate into the epileptic rat hippocampal network by forming afferent and efferent synaptic connections with the host cells. Furthermore, we show that these cells inhibit epileptiform discharges in hippocampal slices in vitro when activated optogenetically and reduce the frequency of motor seizures in chronically epileptic rats in vivo.

The rationale for transplanting GABAergic neurons, derived from different sources, into the epileptic brain is based on an assumption that seizures arise due to increased excitability of neuronal circuits caused by an imbalance between excitatory and inhibitory synaptic processes. This imbalance is thought to be a consequence of the impairment of GABAergic synaptic transmission as a consequence of interneuron degeneration documented in a number of studies [18,19,20]. Thus, supplementing GABAergic interneurons by transplantation is supposed to ameliorate the impaired excitability and thereby counteract seizures. Indeed, several animal studies have demonstrated the beneficial effects of grafting foetal GABAergic neuron precursors in the epileptic hippocampus [21,22,23,24,25,26]. Although proving the principle in experimental conditions, foetal progenitors have limited if any translational value and cannot be applied clinically due to ethical concerns and variability in the quality of cell sources. A more viable source of cells for clinical application is hPSCs and their differentiation into MGE-like cells. Transplantation of these cells has proven to inhibit SRSs and behavioural comorbidities in various TLE models [27,28]. From the clinical perspective, a potential limitation of the approach used in these studies is a relatively slow differentiation rate of the cells taking five or even seven weeks to be ready for grafting. In our study, we transplanted hESC-derived neuronal precursors after only seven days in culture, while overexpressing *Ascl1* and *Dlx2*, two transcription factors necessary for determining their GABAergic fate [30]. Importantly, already at this early time point, the cells expressed neither stem cell nor mitotic markers, thus decreasing the risk of tumour formation (Figure S3). This short and simple protocol gives a significant advantage in terms of sustainability, lower demand on resources, and relatively high reproducibility [29,31].

Our present study demonstrates the maturation of hESC-derived interneurons over time from three months up to six-months after transplantation. All recorded cells were able to generate APs, with the properties maturing over time, reflecting corresponding increases in voltage-dependent sodium and potassium currents. Moreover, spontaneous synaptic activity was increased at the later timepoint PT, indicating improved integration into the neural network, by receiving more afferent synapses. Importantly, we demonstrate that the transplanted cells form efferent inhibitory synapses onto the host neurons providing a possibility of increased GABA release within the hippocampal network. In previous studies, synaptic integration of hPSC-derived interneurons into the epileptic rodent hippocampus was reported at five months PT histologically [28]. Functional maturation and efferent synapse formation of grafted hPSC-derived interneurons were also shown previously [27] although authors did not investigate whether these efferent connections were increasing over time. In yet another recent study authors report electrophysiological and morphological maturation of transplanted hPSC-derived interneurons from 16 to 24 weeks PT, however, without studying functional efferent synapse formation [32]. These cells failed to suppress SRSs in a mouse TLE model [33]. In our study, we report the continuing maturation of the grafted hdInts from three- to six-months PT with an increase in efferent synapse formation over time.

Interestingly, the time range of the delayed graft-derived synaptic responses, measured from the switch-on of the light illumination of the slices, was quite broad (Figure S5). Several factors may contribute to such variation (Figure S6). One possible explanation could be diverse wiring of the connections, ranging from direct synaptic input from a grafted hdInt to the patched cell, to a multisynaptic pattern with a variable number of intermediate neurons activated by the hdInt efferents before the patched cell is finally responding, which would explain the long delay times. The latter scenario is only possible when assuming altered chloride homeostasis in the epileptic tissue converting GABA from hyperpolarising to a depolarising excitatory neurotransmitter [34,35] or as a consequence of neuronal damage induced during the slice preparation [36]. In addition, the longer delay times can also be a consequence of a delayed light-induced AP onset in the optogenetically stimulated hdInts (Figures S6B and S7), presumably depending on a variable level of ChR2 expression within the hdInt population in different cells.

Apart from the GABAergic phenotype of the grafted cells assessed by electrophysiology and immunohistochemistry, the predominant subtypes of the grafted hdInts were consistent with those expressing CR and CB as was also the case in our in vitro study [29]. Unfortunately, around 30% of the grafted cells remained unidentified, due to the lack of sufficient hippocampal sub-slices remaining after electrophysiological experiments for immunohistochemistry staining. Thus, there is a possibility that other subtypes of interneurons, such as somatostatin-, parvalbumin- or neuropeptide Y-expressing interneurons have been missed in these 30%. Nevertheless, one could argue that the presence of CR and CB interneurons observed in the grafts could be sufficient for a beneficial effect, since it has been shown for example that in the human epileptic hippocampus CB interneurons display an altered morphology, and the number of CR interneurons is significantly reduced [37]. Additionally, in rodent epilepsy models, CR interneurons appear to be vulnerable to excitotoxic damage [38]. In line with these observations, a recent study with reprogrammed glial cells into predominantly CR interneurons using the same transcription factors reported a reduction in chronic SRSs in a mouse model of epilepsy [39].

One obvious question regarding our study is whether video monitoring (without EEG recordings) for one week is sufficient as a reliable outcome measure of the effect of the grating of hdInts on SRSs. As mentioned previously in Section 2.1, when animals were monitored with both EEG and video, on average 97% of the SRSs were generalised, with clear motor components that were similar to those analysed in the experimental cohort with only video monitoring (Figure S2). Moreover, it has been reported in a similar KA-SE rat TLE model that 94% of all seizures detected by combined video-EEG monitoring were stage 5, thus detectable on video [28]. In the same study, grafting of hPSC-derived interneurons resulted in a 72% decrease of motor SRSs over a three-week recording period at the fifth month after SE. Importantly, this seizure-suppressing effect remained stable during the course of these 3 weeks. The authors also reported a significant reduction in total time spent in seizures, while no difference in the average duration of individual seizures was observed [28]. Taken together, these data support our assumption that the observed decrease (87%) in motor SRSs in our study reflects the alterations in almost all seizures that these animals experienced. However, we cannot exclude that transplanted hdInts may have converted motor SRSs into milder, only electrographic non-generalised SRSs. Even if this would be the case, this result on its own could be considered as a major positive outcome of the treatment.

In conclusion, our new data provide proof-of-concept of seizure-suppressant effects of grafted hdInts generated by a simple, fast, and efficient protocol for interneuron differentiation. This protocol proved to provide a reliable and renewable source of hdInts showing positive outcomes on various epileptic phenotype read-outs, including optogenetic inhibition of epileptiform discharges in vitro and SRSs in vivo. Although certain aspects of hdInt transplantation, such as more detailed histological analysis and EEG characterisation, need further investigation, this study can be considered as an important milestone in the development of a cell-based therapy for treating drug-resistant epilepsy.

## 4. Materials and Methods

### 4.1. Animals

Immunodeficient nude rat males (RNU rat, Charles River, Wilmington, MA, USA) were housed under a 12/12-h light cycle with ad libitum access to water and food in individually ventilated cages. A total of 25 rats were used.

The experimental procedures performed were approved by the local ethical committee for experimental animals (Ethical permit no. M47-15 and M49-15) and conducted in agreement with the Swedish Animal Welfare Agency regulations and the EU Directive 2010/63/EU for animal experiments.

### 4.2. Lentiviral Constructs and Virus Generation

The following lentivirus constructs were used: lentivirus vector hSyn-hChR2(H134R)-mCherry-WPRE (obtained by cloning at the lab from Addgene #20945, a gift from Karl Deisseroth [40]) for expressing channelrhodopsin-2 (ChR2) coupled with mCherry; and for the doxycycline-inducible Tet-On system: lentivirus vector FUW-TetO-Ascl1-T2A-puromycin (Addgene #97329) for expressing Ascl1-T2A-puromycin cassette; lentivirus vector FUW-TetO-Dlx2-IRES-hygromycin (Addgene #97330) for expressing Dlx2-IRES-hygromycin cassette; and lentivirus vector FUW-rtTA (Addgene #20342) containing rtTA, all gift from Marius Werning [30]. The lentiviral particles were produced as described elsewhere [41].

### 4.3. Cell Culture

H1 (WA01) ES cells were obtained from WiCell Research Resources (WiCell, Madison, WI, USA). hESCs were maintained as feeder-free cultures in mTeSR1 medium (Stem Cell Technologies, Vancouver, BC, Canada) and Matrigel-coated plates (Corning, Corning, NY, USA).

#### 4.3.1. Generation of Induced GABAergic Interneuron Precursors from hESCs

H1 ESCs were transduced with Tet-On system lentiviral particles and ChR2-mCherry lentivirus. Cells were then expanded as needed, frozen down, and kept at −150 °C as a stock for starting differentiation.

The induced GABAergic interneurons were generated as described in Gonzalez-Ramos et al., 2021. Briefly, Tet-On-ChR2-H1 ESCs were thawed and expanded as needed. For starting the differentiation protocol, 60–70% of confluent cells were treated with Accutase (Stem Cell Technologies, Vancouver, BC, Canada) and plated as dissociated cells in six well plates (~3–5 × 10^5^ cells/well) on day 0. Cells were plated on plates coated with Matrigel (Corning, Corning, NY, USA), in mTeSR1 containing 10 mM Y-27632 (Stem Cell Technologies, Vancouver, BC, Canada). On day 1, the culture medium was replaced with N2 Medium consisting of DMEM/F12 supplemented with 1% N-2 Supplement (both Gibco, Waltham, MA, USA), containing doxycycline (2 g/L, Sigma Aldrich, St. Louis, MO, USA) to induce Tet-On gene expression. The culture was retained in the medium for one week. On day 2, a drug-resistance selection period was started by adding puromycin (0.5 mg/mL, Gibco, Waltham, MA, USA) and hygromycin (750 mg/mL, Invitrogen, Waltham, MA, USA) to the fresh media. On day 4, the medium was replaced containing all antibiotics and on day 5, antibiotics were removed and cytosine β-D-arabinofuranoside (Ara-C, 4 µM, Sigma Aldrich, St. Louis, MO, USA) was added. On day 7, cells were used for in vivo grafting.

#### 4.3.2. Cell Transplantation

Cell transplantation was performed four weeks post SE. Neuronal precursors were dissociated at 7 DIV using Tryple Express Enzyme (Gibco, Waltham, MA, USA), centrifuged, resuspended to a concentration of 100.000 cells/uL in HBSS (Gibco, Waltham, MA, USA) containing 10 mM Y-27632 and DNase I Solution (1 µg/mL, Stem Cell Technologies, Vancouver, BC, Canada) and kept on ice until grafting. Cells were then injected stereotaxically in the hippocampus of epileptic RNU rats under isoflurane anaesthesia. Cells were injected bilaterally in both hippocampi with the following coordinates from bregma: anterior-posterior (AP) −6.2 mm, medial-lateral (ML) ±5.2 mm, dorsal-ventral (DV) −6.0, −4.8 and −3.6 mm, 3 μL in total per hippocampus (1 μL at each DV coordinate). Animals were given doxycycline in drinking water (1 mg/mL, 0.5% sucrose) for two days before and four weeks PT to continue the cell differentiation in vivo. Cells remaining after grafting were re-plated on Matrigel-coated coverslips in 24-well plates and cultured in N2 medium overnight, until being fixed with 4% paraformaldehyde containing 0.25% glutaraldehyde and used for immunocytochemistry.

### 4.4. Induction of Status Epilepticus

Male immunodeficient RNU rats (7–8-week-old) were injected subcutaneously in the neck region with an initial dose of 10 mg/kg of KA and subsequently with 5 mg/kg every hour until the first stage 3 or higher seizure grade was observed (scheme of the process is illustrated in Figure S1). Seizures were classified according to the modified Racine scale registering only stages 3 and higher: (3) unilateral forelimb clonus; (4) generalized seizure with rearing, body jerks, bilateral forelimb clonus; (5) generalized seizure with rearing, imbalance, falling, or wild running [42] (Video S1). SE was defined as at least four seizures per hour. After SE, the animals were injected with a Ringer/glucose (25 mg/mL) solution (1:1 ratio) and returned to housing cages. Animals were weighed every day for a week after SE induction and subsequently once per week. Cell transplantation was performed four weeks after SE induction.

### 4.5. Video Recordings and Video-EEG Recordings

To assess the frequency of motor SRSs, animals were video monitored continuously for four months after SE induction. During the dark (night) hours, infrared lamps were used to illuminate the cages. Videos were manually analysed retrospectively and animals not showing SRSs were excluded from the study. Only motor SRSs were detected, noting the time of seizure occurrence, duration of the seizure, and seizure severity (Video S2). Duration of seizures and seizure severity was averaged for each animal, seizure frequency was calculated as a number of seizures per hour for each animal and these values were then used for statistical analyses.

Furthermore, three non-grafted animals underwent implantation of electrodes and transmitters for wireless video-EEG monitoring five months after SE induction. This was done to determine if this rat strain tolerates the procedure and the implants and for further characterisation of their seizures. The whole procedure was performed as described previously [43]. Firstly, the rats were anesthetized with 4% isoflurane and placed in the stereotaxic frame while kept on 2% isofluorane. The transmitter (F40-EET, Data Sciences International, St. Paul, MN, USA) was placed in a subcutaneous pocket on the rats’ backs. One stainless steel electrode (Plastics One, Roanoke, VA, USA), soldered to the wire of the transmitter, was implanted at the following coordinates: AP −6.2 mm, ML +5.2 mm, DV −6.0 mm. The second electrode was placed on top of dura mater above the motor cortex ipsilateral to the depth electrode. Two reference electrodes were placed on the dura mater, 2 mm rostral to the lambda. Two stainless screws were attached to the skull bone to secure the electrode assembly by dental cement. Animals were weighed every day for a week after implantation and consequently once per week henceforth. To begin the video-EEG monitoring, the wireless transmitter was activated by a magnet and the cage was placed on top of a receiver unit (Data Sciences International, St. Paul, MN, USA). Two cameras (Axis, Lund, Sweden) were used to continuously record video of the animal activity for 30 h, and seizures were then detected off-line in NeuroScore (Data Sciences International, St. Paul, MN, USA).

### 4.6. Electrophysiology

#### 4.6.1. Whole-Cell Patch-Clamp Recordings in Hippocampal Slices

RNU rats at three- or six-months PT were briefly anesthetized with isoflurane and decapitated. Brains were transferred to an ice-cold modified artificial cerebrospinal fluid (aCSF) solution containing in mM: 75 sucrose, 67 NaCl, 26 NaHCO_3_, 25 D-glucose, 2.5 KCl, 1.25 NaH_2_PO_4_, 0.5 CaCl_2_, 7 MgCl_2_ (all from Sigma Aldrich, St. Louis, MO, USA), equilibrated with carbogen (95% O_2_/5% CO_2_), with pH 7.4 and osmolarity ~300 mOsm. The brains were cut on a vibratome (VT1200S, Leica Microsystems, Wetzlar, Germany) into 300 μm thick quasi-horizontal hippocampal slices, which were transferred to aCSF containing in mM: 118 NaCl, 2 MgCl_2_, 2 CaCl_2_, 2.5 KCl, 26 NaHCO_3_, 1.25 NaH_2_PO_4_, 10 D-glucose. Slices were incubated in this solution for 30 min at 34 °C, and subsequently at room temperature until recordings were performed. The individual cells in the slices were visualized for whole-cell patch-clamp recordings using infrared differential interference contrast video microscopy (BX51WI; Olympus, Shinjuku, Tokyo, Japan). Recordings were performed from grafted (identified under fluorescence with 520 nm light for mCherry+) and host cells (mCherry−) at 32 °C using a glass pipette filled with a solution containing (in mM): 140 KCl, 10 NaCl, 10 HEPES, 0.2 EGTA, 4 MgATP, and 0.4 Na_3_GTP (~300 mOsm, pH 7.2; all from Sigma Aldrich, St. Louis, MO, USA). This solution inverts the polarity of chloride currents inward while increasing their amplitude, making them easier to detect. Average pipette resistance was between 2 and 4 MΩ, pipette capacitance was compensated for during cell-attached configuration. Biocytin (0.5–1 mg/mL, Biotium, Fremont, CA, USA) was included in the pipette solution to retrospectively identify recorded cells. All recordings were done using a HEKA EPC10 amplifier (HEKA Elektronik, Lambrecht, Germany) and sampled at 10 kHz with a 3 kHz Bessel anti-aliasing filter and using PatchMaster software for data acquisition.

#### 4.6.2. Passive Membrane Properties of Transplanted Cells

Estimated resting membrane potential (RMP), series resistance (Rs), input resistance (Ri), and cell membrane capacitance (Cm) were calculated from a series of 5 mV pulses of 100 ms duration, applied through the patch pipette immediately after whole-cell configuration was established. The membrane capacitance was calculated from the charge integration of the transient response to the test pulse. To determine the ability to fire action potentials (APs), 500 ms current steps ranging from −40 pA to 200 pA in 10 pA steps were applied while holding the cell membrane potential at approximately −70 mV. From the same holding potential, 1-s linear ramp currents were injected into the cells to determine the AP threshold. AP amplitude was measured from threshold to peak, and duration was measured as the width at the threshold. The amplitude of the afterhyperpolarization (AHP) was measured as the difference between the AHP peak and the AP threshold. Sodium and potassium currents were evoked by a series of 100 ms long voltage steps ranging from −90 mV to +40 mV in 10 mV steps. In addition, their sensitivity to 1 µM tetrodotoxin (TTX, Abcam, Cambridge, UK) and 10 mM tetraethylammonium (TEA, Abcam, Cambridge, UK) was assessed.

#### 4.6.3. Optogenetics

For optogenetic depolarization of ChR2-expressing cells, blue light was applied at 460 nm wavelength with a LED light source (Prizmatix, Holon, Israel) and delivered through a water immersion 40× microscope objective. Blue light was delivered for a duration of 500 milliseconds, or by 5 pulses of 3 milliseconds repeated at 10 Hz. For detection of graft-to-host synaptic connections, the same stimulation was used during recoding from a “host” cell in the vicinity of a ChR2-expressing cell.

#### 4.6.4. Spontaneous Synaptic Activity

Spontaneous postsynaptic currents (sPSCs) were recorded at −70 mV. Whole-cell patch-clamp recordings of sPSCs were analysed offline with Igor Pro (Wavemetrics, Portland, OR, USA) and Python. sPSCs were detected automatically and analysed using a custom Python script [44]. A postsynaptic current template was generated from the voltage-clamp recordings which were low-pass filtered at 400 Hz and was used for the detection algorithm. Events with a correlation coefficient to the template lower than 0.6 were excluded from the analysis, as well as those with amplitude <3 pA and rise-time >5 ms. For distribution comparisons, an equal number of events was analysed from all recorded neurons, while for median comparisons all events were considered.

#### 4.6.5. Drugs and Concentrations

For the blocking of GABAA and GABAC receptors, picrotoxin (PTX, 100 μM, Tocris, Bristol, UK) was used, although it might act on glycine and 5-HT_3_ receptors [45,46]. However, the used concentration of PTX is considered to be insufficient to block the signalling through serotonin receptors [47]. (2R)-amino-5-phosphonovaleric acid (D-AP5, 50 μM, Abcam, Cambridge, UK) and 2,3-dihydroxy-6-nitro-7-sulfamoyl-benzo-quinoxaline-2,3-dione disodium salt (NBQX, 10 μM, Alomone Labs, Jerusalem, Israel) were used to block NMDA and AMPA receptors, respectively. TTX (1 μM, Abcam, Cambridge, UK) and TEA (10 mM, Abcam, Cambridge, UK) were used to block sodium and potassium channels, respectively.

#### 4.6.6. Epileptiform Activity and Local Field Potential Recordings

To test the effect of grafted hdInts on epileptiform activity in vitro, we used local field potential (LFP) recordings in hippocampal slices from six-month PT rats. The slices were perfused by either high-K^+^ aCSF or zero-Mg^2+^ aCSF. The high-K^+^ aCSF contained in mM: 118 NaCl, 2 MgCl_2_, 2 CaCl_2_, 26 NaHCO_3_, 1.25 NaH_2_PO_4_, 10 D-glucose and 7.5 to 9.5 KCl. The zero- Mg^2+^ was the same but contained 2.5 KCl and no MgCl_2_. LFPs were recorded by a pipette of 1–3 MΩ resistance filled by the same aCSF. The pipette was placed in the vicinity of the graft and where the epileptiform discharges were most prominent. The LFPs were amplified and sampled at 10 kHz. To assess whether hdInts could suppress the epileptiform discharges, we activated them by blue light stimulation. We tested 3 stimulation protocols separated by 2 min of baseline (no light): (1) Five-second continuous light pulse separated by 35 s of a dark period, repeated 30 times. (2) Five-second pulse train at 30 Hz and 3 ms pulse width separated by 35 s of a dark period, repeated 30 times. (3) Three-minute pulse train at 30 Hz and 3 ms pulse width.

The epileptiform discharges were detected and analysed offline using a custom-made script in Matlab. Briefly, the signal was filtered between 2 and 400 Hz, comb-filtered to remove power line noise, and down-sampled to 1 kHz. Then, the signal power was computed in a 50 ms window, sliding sample-by-sample. The threshold for detection was determined as $4\times median\times \sqrt{kurtosis}$ of the power and detections were marked at the maxima of the regions exceeding the threshold.

We analysed the frequency of occurrence of the events and, using the filtered signal, we computed 3 parameters for each event: peak-to-peak amplitude, peak signal power, and coastline index according to the formula: $\mathrm{Coastline}=\frac{1}{N}\sum_{n=1}^{N-1}\left|y\left(n+1\right)-y\left(n\right)\right|$, where *y* is the filtered signal, *N* is the number of samples and *n* is the index of the sample. For the statistical analysis, the mean of these parameters was computed for the dark period and light stimulation period in each of the 30 runs of the given protocol in the given slice. Then, the runs were averaged and the resulting two numbers per slice (dark and light) were subtracted. The differences were then evaluated by Wilcoxon signed-rank test.

### 4.7. Immunohistochemistry, Immunocytochemistry, Imaging, and Quantification

For immunohistochemistry, 300 μm slices used for electrophysiological experiments were collected from aCSF and immediately fixed in 4% paraformaldehyde containing 0.25% glutaraldehyde. After overnight fixation at 4 °C, slices were transferred to 20% sucrose in 0.1 M sodium phosphate-buffered saline and kept at 4 °C for at least two days until further processing. The slices were either immediately stained or cut on a microtome into 30 μm thick sections and stored in a glycerol-based antifreeze solution at −20 °C until stained. For staining, sections were washed thoroughly with PBS, then blocked in 5% serum of the species specific to the secondary antibody, in PBS containing 0.25% Triton-X and incubated with primary antibodies overnight (or for 48 h for 300 μm slices) in the same solution at 4 °C. Following primary antibody incubation, sections were washed with PBS and blocked again with the same serum solution as above. Then, sections were incubated with secondary antibodies for 2 h (or 24 h for 300 μm slices) either fluorophore-conjugated to allow for fluorescent detection (AlexaFluor Plus 488/555/647, 1:1000, Thermo Fisher Scientific, Waltham, MA, USA), or biotinylated for streptavidin amplification (1:200, Vector Laboratories, Burlingame, CA, USA). In some cases, for signal amplification and for visualisation of patched biocytin-filled cells, streptavidin-conjugated fluorophores were used (1:2000, Jackson Immunoresearch, West Grove, PA, USA). Immunofluorescent sections were coverslipped with PVA-DABCO containing 1:1000 Hoechst. For detailed information of antibodies and dilutions used, see Table S2. Images were acquired either by confocal microscopy (Nikon Confocal A1RHD microscope, Nikon, Minato, Tokyo, Japan) or by epifluorescence microscopy (Olympus BX61, Olympus, Shinjuku, Tokyo, Japan). For immunocytochemistry, the same staining process was used, omitting the second-day blocking step.

For all quantifications ImageJ software (NIH, Annapolis, MD, USA) was used. For quantification of GABA immunostaining, mean fluorescence intensity was measured in confocal Z-stack images taken at 20× magnification using maximal intensity projection from 13 stacks (1 µm distance). In each slice, the area of the graft was outlined based on the STEM121 immunostaining, and the median grey value was measured in the GABA-staining channel. Together with the graft area, an area outside of the graft was outlined and fluorescence intensity was measured. The two values in each slice were then compared. For quantification of CR and CB stainings, similar Z-stack images were composed. Firstly, all cells positive for the human cytoplasm marker (STEM121+ cells) were counted, and Hoechst was used to visualise the nuclei and identify individual cells. Secondly, cells double positive for STEM121 and CB or CR, respectively, were counted. A percentage of double-positive cells out of all STEM121+ cells was calculated for each slice and used for statistical analyses.

### 4.8. Statistical Analyses

Statistical analysis of the data was performed using Prism 9 software (GraphPad, San Diego, CA, USA). A Mann-Whitney test was used for comparison of medians, an unpaired *t*-test was used for comparison of means when data were normally distributed, the Wilcoxon test was used for paired data, and the binomial test was used for comparison of proportions. Spearman correlation was used for exploring the relationship of two variables. The level of significance for these tests was set at *p* < 0.05. The Kolmogorov-Smirnov test was used for distribution comparisons and the level of significance was set to *p* < 0.01 and *D* > 0.1. In box plots, the centre line represents the median, the box edges represent interquartile range, and whiskers the complete range of the values. The individual values are plotted as dots. In the rest of the graphs, the mean ± SEM is shown, which is also used to represent values in the main text.

## Data Availability

Data used for this study is available on request from the corresponding authors.

## Supplementary Materials

The following are available online at https://www.mdpi.com/article/10.3390/ijms222413243/s1.
