# Supplementary material for: Human Stem Cell-Derived GABAergic Interneurons Establish Efferent Synapses onto Host Neurons in Rat Epileptic Hippocampus and Inhibit Spontaneous Recurrent Seizures
Source: Int J Mol Sci. 2021 Dec 8;22(24):13243. doi: 10.3390/ijms222413243 (PMC8705828; doi:10.3390/ijms222413243)
Supplement: Supplementary file 1 [file ijms-22-13243-s001.zip › Supplementary/Supplementary Materials.pdf]

Transplanted human stem cell-derived GABAergic interneurons establish efferent synapses with host neurons in rat epileptic hippocampus and inhibit spontaneous recurrent seizures

Supplementary materials

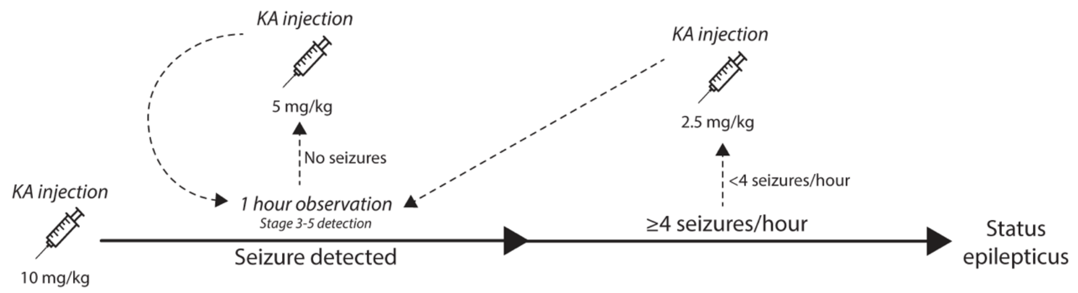

Figure S1. Schematic illustration of SE induction by KA. Rats were first injected with 10 mg/kg of KA, followed by a 1-hour observational period. If a stage 3 or higher seizure grade was detected, the rats were observed for another hour to determine the seizure frequency. If at least 4 seizures/hour were observed, rats were considered as having SE. If after the first KA injection, the rats did not develop seizures within an hour, a 5 mg/kg KA injection was added with the follow-up observation time as before. If during the assessment of seizure frequency less than 4 seizures/hour were observed, a 2.5 mg/kg dose of KA was added, followed by the same observation time until reaching the desired number of at least 4 generalised seizures per hour.

Video S1. Example of seizures detected during SE induction. Rat on the left displays a stage 5 seizure with rearing, loosing balance and falling. Rat on the left displays a stage 4 seizure with rearing, body jerks and bilateral forelimb clonus.

Video S2. Example of seizures detected during video-recordings 4 months post SE. (**A**) Rat #7 (lower right) displays a stage 5 seizure with rearing, loosing balance and falling. (**B**) Rat #6 (lower left) displays a stage 5 seizure with rearing, loosing balance and falling. (**C**) Rat #6 (lower left) displays a stage 4 seizure with rearing, body jerks and bilateral forelimb clonus.

Table S1. Summary of primary antibodies and dilutions used.

ANTIBODY	HOST	COMPANY	CAT. NO.	DILUTION
mCherry	Chicken	Abcam	Ab205402	1:2000
GABA	Rabbit	Sigma Aldrich	A2052	1:2000
Calbindin	Rabbit	Swant	CB-38a	1:1000
Calretinin	Rabbit	Swant	CR-7697	1:1000
STEM121*	Mouse	Takara Bio	Y40410	1:400

Sox2	Rabbit	Abcam	Ab97959	1:1000
Ki67	Rabbit	Novocastra	NCL-Ki67p	1:250
Nestin	Mouse	Abcam	Ab6142	1:500
β-III-tubulin	Rabbit	Abcam	Ab18207	1:1000

*Streptavidin amplification was used for immunofluorescence

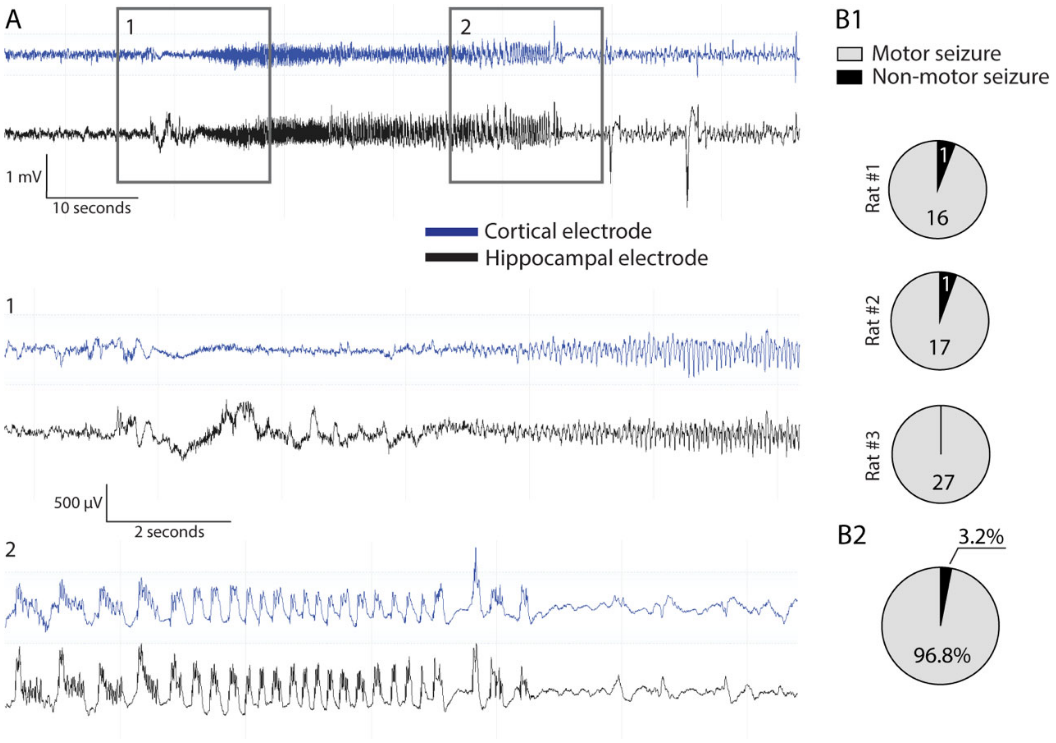

Figure S2. Electrographic characterisation of SRSs in KA induced epileptic rats 4-months post SE induction. (**A**) Representative EEG trace of a SRSs. Magnification 1 shows the beginning of the seizure, illustrating its origin in the hippocampus and the spread to the motor cortex. Magnification 2 indicates the end of the seizure and return to normal EEG activity. (**B1**) Quantification of the proportion of motor SRSs out of all detected electrographic SRSs in 3 rats. (**B2**) Pooled data from rats in B1 represented as percentages. Vast majority of all SRSs were generalised motor seizures.

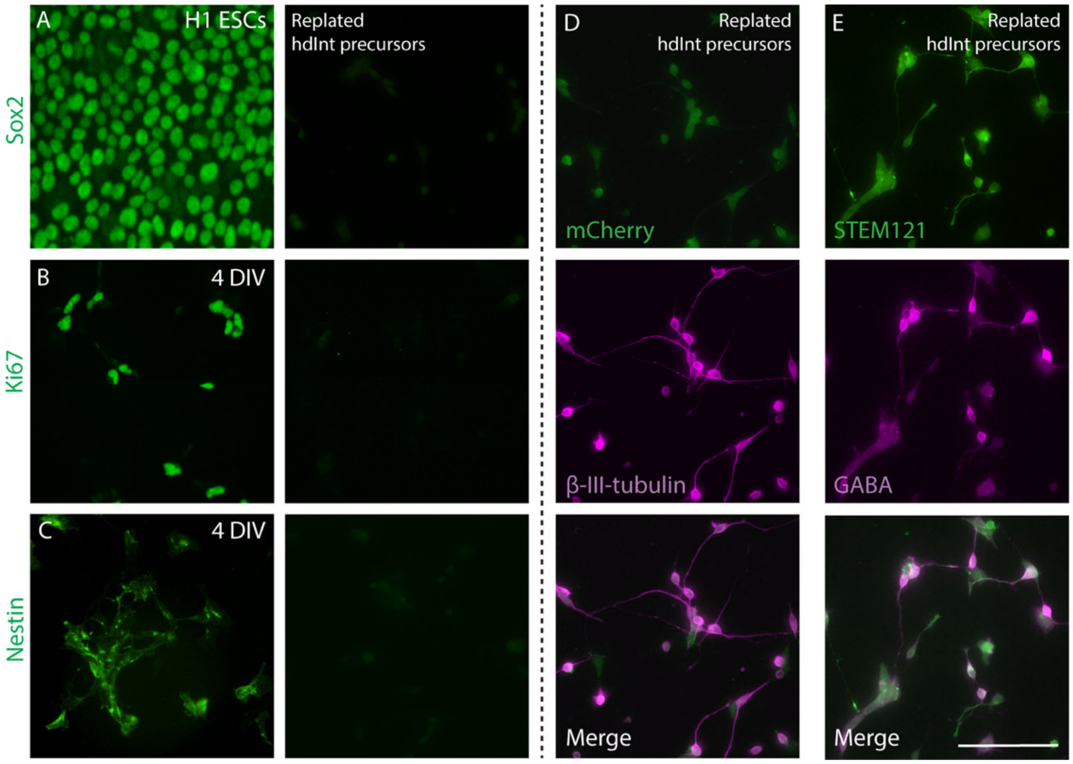

Figure S3. Stainings of left-over hdInt precursor cells remaining after grafting. (**A**) At time of transplantation hdInt precursors did not express Sox 2; H1 ESCs were used as a positive control. (**B, C**) hdInt precursors at time of transplantation did not express Ki67 nor Nestin. 4 DIV hdInt precursors were used as a positive control. (**D**) hdInt precursors expressing mCherry (in green) and β-III-tubulin (in magenta) at the time of transplantation. (**E**) Staining of hdInt precursors with STEM121 (in green), a human cytoplasm marker, and GABA (in magenta). Scale bar 100 μm.

Table S2. Electrophysiological properties of grafted hdInts. Table comparing the electrophysiological properties of grafted cells at 3 months (n = 26 cells) and 6 months (n = 42 cells) PT regarding their response to blue light, their intrinsic properties, and properties of recorded spontaneous postsynaptic events. Values are represented as mean ± SEM. Binomial test was used to compare proportions, Mann-Whitney test for comparison of medians. *, p<.05; **, p<.01; ****, p<.0001. AP, action potential; Ri, input resistance RMP, resting membrane potential; Cm, membrane capacitance; Rs, series resistance; AHP, afterhyperpolarization.

Light response properties

	3 months PT	6 months PT
APs to light	37%	57%**
Peak current (pA)	115.6 ± 20.97	80.52 ± 13.98
Steady state current (pA)	50.23 ± 10.3	41.5 ± 7.9

Intrinsic properties

	3 months PT	6 months PT
Ri (MW)	974 ± 75.23	746.4 ± 62.4*
RMP (mV)	-52.64 ± 1.86	-59.77 ± 1.26**
Cm (pF)	188.7 ± 24.01	205.8 ± 16.37
Rs (MW)	9.91 ± 1.35	8.48 ± 1.08
AP amplitude (mV)	54.56 ± 3.53	76.79 ± 1.95****
AP threshold (mV)	-31.82 ± 1.62	-36.5 ± 1.05*
AP duration (ms)	3.35 ± 0.32	2.04 ± 0.13****
AHP amplitude (mV)	22.28 ± 1.06	28.39 ± 0.91****

Properties of spontaneous postsynaptic currents

	3 months PT	6 months PT
Rise time (ms)	2.0 ± 0.06	1.69 ± 0.04****
Amplitude (pA)	8.14 ± 0.77	15.32 ± 1.6****
Frequency (Hz)	1.36 ± 0.24	2.52 ± 0.2****

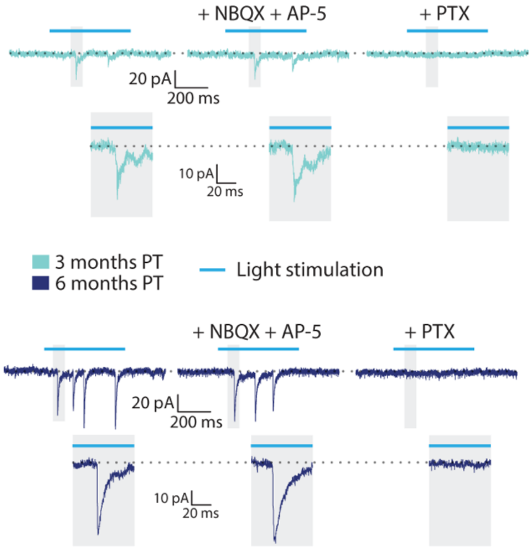

Figure S4. GABAergic nature of hdInt-mediated efferent synaptic connections. Voltage-clamp recordings from patched host cells at 3 months and 6 months PT. Blue light illumination of the slices resulted in delayed synaptic responses which were not affected by NBQX and AP-5 application, while were completely blocked by PTX.

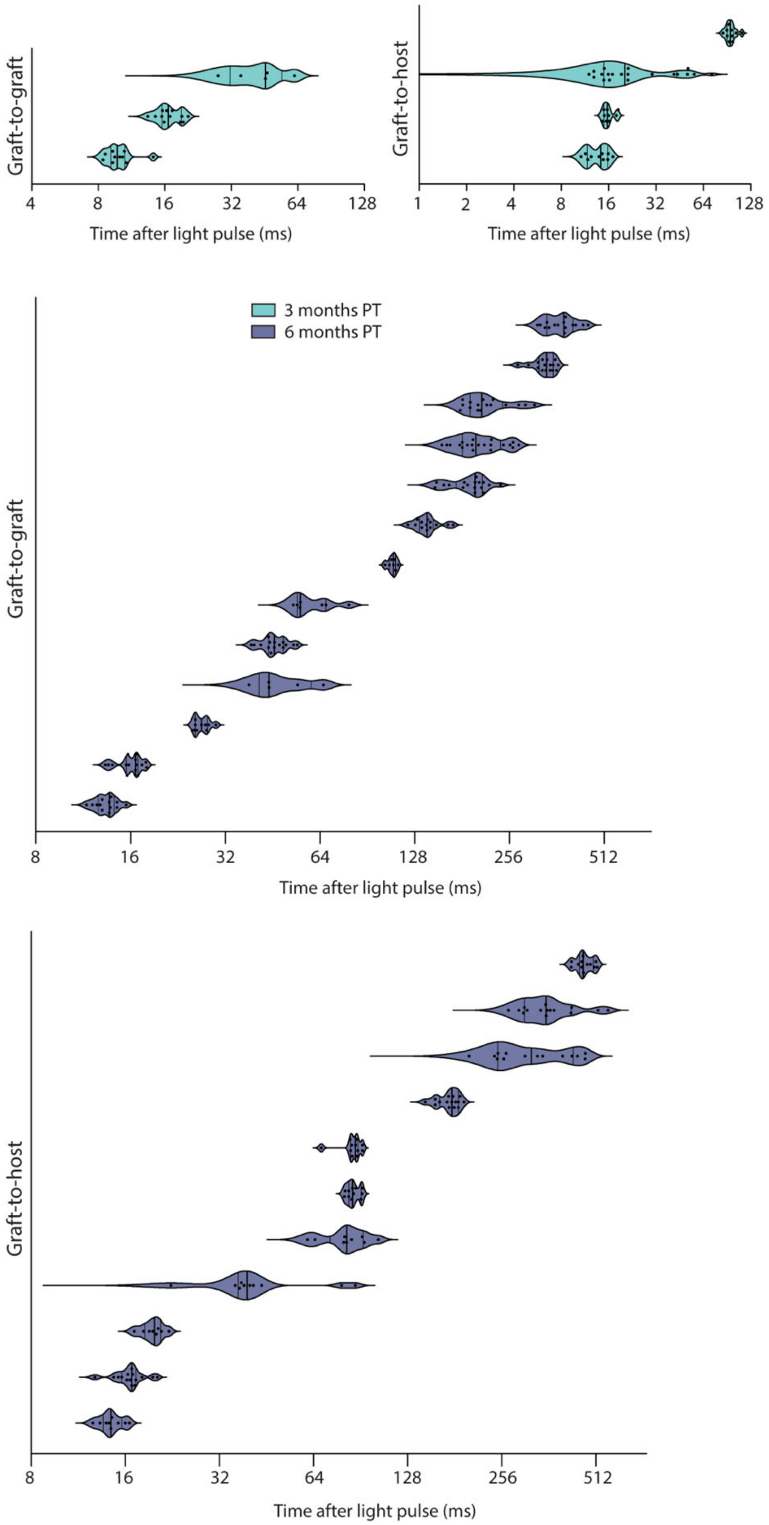

Figure S5. Individual light-induced delayed synaptic responses from each recorded cell. The time was measured from the beginning of the 500 ms light pulse to the base of the first synaptic event afterwards. Each violin plot represents an individual cell, dots are individually measured delays from each recorded trace. Thicker midline represents the median and thinner lines the interquartile range. Time axis is in exponential scale.

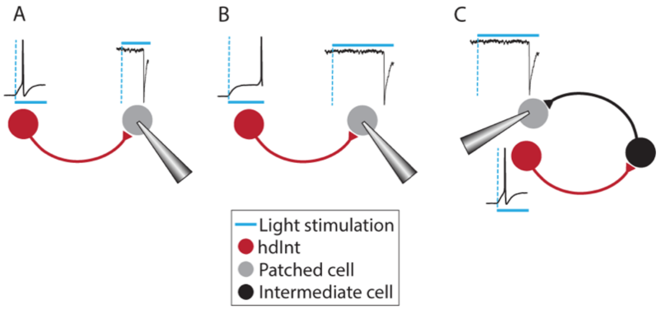

Figure S6. Cartoon of possible synaptic connections between grafted presynaptic cells and patched cells. (**A**) A direct connection between 2 cells with a short light-induced AP onset of the grafted hdInt resulting in a short delayed synaptic response in the patched neuron. (**B**) A direct connection between 2 cells with a delayed light-induced AP onset of the grafted hdInt resulting in a longer delay in the synaptic response in the patched neuron. (**C**) A multi-synaptic connection resulting in a longer delay in the synaptic response of the patched cell.

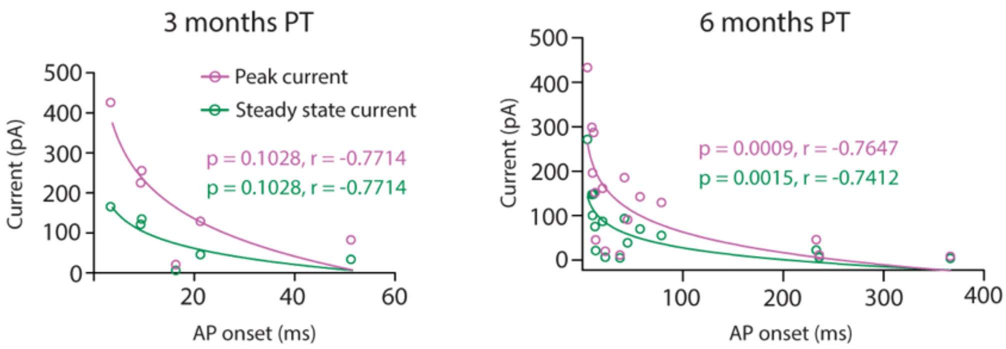

Figure S7. Light-induced currents of grafted hdInts correlated to the light-induced AP onset. The time was measured from the beginning of the 500 ms light pulse to the threshold of the first action potential. Only cells with no observed graft-to-graft connection were analysed. Correlation was not confirmed at 3 months PT (n = 6 cells) but was observed at 6 months PT (n = 16 cells) between both the peak and the steady state current and the AP onset. The higher the current the shorter AP onset. Non-parametric Spearman correlation was used, semilog line was fit in the graphs.
